# Bifactor model of the CASP-12’s general factor for measuring quality of life in older patients

**DOI:** 10.1186/s41687-018-0078-x

**Published:** 2018-12-04

**Authors:** Matthew J. Kerry

**Affiliations:** Zurich University of Applied Sciences (ZHAW) - Institute of Health Sciences, Technikumstrasse 71, 8041 Zurich, Switzerland

## To the editor

Patients’ subscores on quality of life (QoL) measures can provide diagnostic information about strengths and weaknesses of respondents’ performance in specific areas. Such diagnostics may help with identification of potential at-risk individuals. Subscores may also help with modifying extant care-treatment programs, particularly those among patient-preferred specific functionalities [1]. The Control, Autonomy, Self-realization and Pleasure (CASP) measure is one, popular QoL measure example with such subscore potential, which will be of focal interest in the current short report [2].

The CASP builds on psychology needs-satisfaction models to emphasize wellbeing across its four titled domains [3]. The shortened version of the original CASP-19 scale, was designed specifically for use in the Survey of Health, Ageing and Retirement in Europe (SHARE) study (CASP-12) [4], representing two combined factors: 1)Control/Autonomy, and 2) Self-realization/Pleasure. Extant psychometric studies of the CASP-12 have been limited by classical measurement approaches. For example, the proposed combination of CASP’s first two subscales for greater stability contradicts the retention of its other, two shorter subscales exhibiting higher internal reliabilities. Also, proposed combining (or, parceling) of items for fitting unidimensional prediction models potentiates further upward-bias from subdomain-criterion relations.

The current short report’s primary aim is to psychometrically inspect the CASP-12 with modern measurement’s item response theory (IRT). This is important, because increasing usage is potentially unproductive due to incomplete inspection of the CASP’s internal psychometric structure, such as general factor strength and substantive multidimensionality [5]. This limits, among other things, the CASP-12’s equating across studies that use different subsets of items, as well as hindering the CASP’s expansion to new items when CASP-12’s core-pool has not been IRT-calibrated. The current study will identify and extending initial findings from SHARE’s older-adult general population and examine CASP-12’s uni- /multi -dimensionality in a patient-specific sample from the Irish Longitudinal Study on Ageing (TILDA) [6].

Since the early, 1990’s days of QoL research, investigators have generally agreed that physical, mental, and social health subdomains are inseparable, that is, QoL is a fairly broad construct [7]. As mentioned in this author’s earlier IRT evaluation of another health measure– “broader constructs are stabilized with broad factors” [8]. As the CASP’s author reassures researchers that “those who simply require a single index” may sum the CASP-12, it is important to first-determine if unidimensional usage in prediction models is reasonably unbiased by ignoring subdomains. As the CASP constructor’s concluded, “…strength of the inter-domain correlations…. confirm our belief that QoL is a unitary phenomenon which is the *product of the interactions between the domains*” [2]. This interpretation of general QoL as-caused by *inter*-domain interactions is important, because it contradicts the commonly accepted second-order CASP model, which hierarchically represents general QoL as causally preceding variation on its four specific domains (control, autonomy, self-realization, pleasure). If, instead, the CASP’s general QoL factor is correctly interpreted as ‘emerging’ from diverse manifestations represented by subdomains, then within-domain variation may be more accurately viewed more-so as nuisance variation that can and should be statistically treated as such in the measurement of QoL [9, 10]. For example, Sexton and others’ have suggested to covary residuals for CASP’s negatively worded items “arising from method effects” [11]. Fitting this alternative view, the bifactor model is a viable competitor to the second-order hierarchical model that will be empirically compared on model-data fit, as well as aligning more closely with CASP’s theoretical conceptualization as a unitary assessment of QoL.

As CASP’s original author, Hyde, recently stated – “It has proven to be a…multidimensional instrument” [9]. The primary aim of the current study is to examine the substantiveness of such multidimensionality, which should be well-admitted in the context of QoL assessment among older patients. The next section details the samples and analyses conducted to report findings from the CASP’s psychometric inspection with IRT [12].

## Methods

### Measurement instrument

The CASP-12 self-report QoL instrument comprises twelve items. Each item is scored on a 4-point Likert-type scale, with descriptive anchors provided for each response option: 1 (‘Often’), 2 (‘Sometimes’), 3 (‘Not often’), and 4 (‘Never’). Higher CASP total-scores (CASP_TOT_) are interpreted as better QoL, with a possible range of: 12–48. In this short report, we denote CASP total-scores as CASP_TOT._ CASP subscales are abbreviated as Control_(Con)_, Autonomy_(Aut)_, Self-Realization_(SR)_, and Pleasure_(Pleas)_; For the CASP-12v.3 model’s two-factor structure examined here, we denote combined subscales as CASP_(Con/Aut)_ and CASP_(SR/Pleas)_, respectively.

### Sample^1^

A retrospective-observational study was conducted using archival data from the Survey of Health, Ageing and Retirement in Europe (SHARE), originally collected with interview methodology. The most recent, cross-sectional SHARE administration of the CASP in SHARE (Wave 6 [W6]) was obtained for current analyses.﻿^1^﻿ Sample1 participants were respondents to the latest cross-section of SHARE’s questionnaire, fielded in 2015. Participants are drawn from a representative sample of community-adults aged > − 50 years, residing in Europe (*N* = 63,669). Sample2 participants respondents to the latest cross-section of TILDA’s questionnaire, fielded in 2015. Participants are drawn from a representative sample of community-adults aged > − 50 years, residing in Ireland(*N* = 4993).

### Analyses

Preliminary analyses, including editing, missingness, and summary statistics were conducted. Latent variable modeling, including item-calibration and model-comparisons was conducted in IRT-PRO v4.1 [13]. Marginal maximum likelihood (MML) estimation with Bock-Aitken expectation-maximization (BA-EM) algorithm was employed for all models. Item parameters and standard errors were estimated using the supplemented-EM algorithm. IRTPRO default values for convergence criteria (E-step = 1e-005; M-step = 1e-006; cycles = 500) and quadrature node details (points = 49; θ range = − 6, 6) were implemented in estimations. As in many IRT-based studies, likelihood-ratio tests were used to test hypotheses.

## Results

SHARE missing values by item ranged from 0.19% (item 1, 10) to 0.95% (item 12), and 97.51% answered all 12 CASP items. TILDA missing values by item ranged from 1.49% (item 11) to 3.42% (item 3), and 91.86% answered all 12 CASP items. The following results were obtained from participants with complete CASP data (*n* = 63,669_SHARE_ / 4993_TILDA_). Summary sample characteristics are displayed in Table 1 below. Univariate item-level descriptive statistics, frequency response patterns, and graphical inspection of normal Q-Q plots provided tentative evidence for inferring univariate-normal distributional assumptions.

**Table 1:** Summary Sample Characteristics

	SHARE W6 Sample		TILDA W3 Sample
Size (n)	*n* = 63,669		*n* = 4993
Age M (SD)	67.68 (10.31)		65.94 (8.58)
Gender			
Male	43%		44%
Female	57%		56%
Marital Status			
Married	69.23%		69.52%
Never Married	5.22%		17.10%
Divorced	7.08%		–
Windowed	18.17%		13.38%

*Note*. Gender and Marital Status values reported as sample proportions (%). Age is reported as sample mean (*M*) with standard deviations (*SD*) in parentheses

Four models of CASP were compared for global fit indices – 1) Unidimensional_(1-DIM)_, 2) CASP-12 v.3’s two-factor _(2-DIM)_, 3) A bifactor with two specific factors specified by the CASP-12 v.3, and 4) Finally, because the combining of factors was aimed at preserving individual-difference indicators on narrower-specific QoL constructs (CASP subdomains), bifactor extension with random-intercepts was added (BiFactor_Rand-Intcpt_) to compare if the content specificity adequately captures idiosyncratic response biases (e.g., careless responding to reverse-score items).

Model-comparisons began with the unidimensional-baseline and currently used CASP-12 v3. model, with the latter and more complex model expectedly fitting better (Δ*χ*^*2*^_[1]_ = 147,456.13*, p < .*001). Consequently, the more complex bifactor model also exhibited significantly better fit than the v3. two-factor structure (Δ*χ*^*2*^_[11]_ = 12,019.17*, p < .*001).

The likelihood ratio comparison between the last-two bifactor models is highly significant (Δ*χ*^*2*^_[1]_ = 1340.61*, p < .*001), suggesting that QoL’s residual dependence is adequately modeled by the simpler bifactor’s specification of CASP item-content subdomains. Comprehensive IRT parameters and associated standard error estimates are reported in Table 2.^2^ Because CASP items are polytomous, with four rating categories, three difficulty thresholds (category intercepts) plus one discrimination parameter (item slope) are shown for each of CASP’s 12 items. The threshold parameters are a cumulative-logit model representing the probability of a person / patient of endorsing that response category, or-any-other higher (Please see Additional file 1).

**Table 2:** Summary Item-Factor Loadings and Comparative Global Model-Fit Indices

	1-Dim	2-Dim		BiFactor ✓			BiFactor_Rand-Intcpt_		
Item		Fac1_C/A_	Fac2_SR/P_	Tot	Fac1_C/A_	Fac2_SR/P_	Tot	Fac1_C/A_	Fac2_SR/P_
1	0.56	0.66	–	0.62	0.27	–	0.5	0.33	–
2	0.53	0.75	–	0.52	0.56	–	0.43	0.63	–
3	0.60	0.81	–	0.54	0.60	–	0.45	0.55	–
4_(r)_	0.57	–	0.56	0.52	–	.23	0.49	−0.01	–
5	0.20	0.35	–	0.12	0.44	–	−0.03	0.31	–
6	−.42	0.46	–	0.37	0.27	–	0.23	0.16	–
7_(r)_	0.73	–	0.75	0.43	–	0.66	0.48	–	0.57
8_(r)_	0.81	–	0.82	0.50	–	0.71	0.56	–	0.61
9_(r)_	0.60	–	0.62	0.35	–	0.56	0.36	–	0.48
10_(r)_	0.77	–	0.77	0.73	–	0.31	0.75	–	0.16
11_(r)_	0.82	–	0.84	0.69	–	0.45	0.72	–	0.3
12_(r)_	0.84	–	0.85	0.69	–	0.47	0.72	–	0.32
*Global Fit*									
*-2lnL*	1,601,460.18	1,586,703.35			1,574,683.58		1,576,024.19		
*Df*	582	581			570		569		
*AIC*	1,601,556.18	1,586,801.35			1,574,803.58		1,576,146.18		
*BIC*	1,601,991.13	1,587,245.36			1,575,347.27		1,576,698.93		
*RMSEA* .05		.05			.04		.04		

Note. N = 63,669. -*2lnL* -2 log likelihood, *AIC* Akaike information criterion, *BIC* Bayesian information criterion. *1-Dim* unidimensional model, *2-DIM* two-dimensional model, *BiFact* Bifactor Models, *Gen* general factor, Fac1, Fac2 = subscale factors. (r) indicates reverse-scored item. One indicates random intercept. All standard errors were < .01

Having identified a bifactor best-fitting model to CASP-12 responses, suggesting retention of the general QoL factor, testing proceeded with inspection of reliability for both CASP_TOT_ and its subscales (can subscales be used?). First and foremost, coefficient alpha (*α*) is not an indicator of unidimensionality and, often, is a poor indicator of reliability [14]. This is verified in our current sample by rejection of tau-equivalency assumptions, ∆*X*^2^(12) = 3462.08, *p* < .01. Instead, the CASP’s item-covariance structure supports congeneric reliability (*ρ*)*,* which protects against coefficient *α*’s underestimation. Here, CASP_TOT_ was estimated as *ρ* = .77. Subscale reliabilities were estimated at *ρ* = .68_(Con/Aut)_ and *ρ* = .84_(SR/Pleas)_.

An alternative reliability index when multidimensionality’s impact is uncertain is coefficient omega (*ω*), which indexes the proportion of variance in CASP_TOT_ scores attributable to all common sources of variance. Here, CASP_TOT_ was estimated as *ω* = .91. Subscale omegas were estimated at *ω* = .77_(Con/Aut)_ and *ω* = .91_(SR/Pleas)_.

We may further index the unique variance after factoring out all other sources of systematic variance. Here, CASP_TOT_ was estimated as *ω*_Hier_ = .83. Consequently, we may subtract *ω*_Hier_ from the previous *ω* value to obtain an estimate of the reliable variance in CASP_TOT_ scores that is due to the subdomains. That is, *ω*(.92) - (.83)*ω*_Hier_ = .09, indicating that 9% of the reliable variance in CASP_TOT_ scores is due to the subdomains. Furthermore, the subscales’ *ω*_Hier_ were estimated at *ω*_Hier_ = .37(Con/Aut), and *ω*_Hier_ = .04(SR/Pleas). These substantially lower values after residualizing-out CASP_TOT_ implies that much of the ‘precision’ inferred from using CASP subdomains as specific QoL constructs is mostly ‘borrowed’ from the reliability of CASP_TOT_’s general QoL factor. This finding is supported by further evidence from Haberman’s 4-step procedure for determining the relative-improvement from using only subscale items to estimate reliability compared to all CASP items. In the current data, lower reliabilities were found for subscale-only items, implying that there is a relative-decrement (rather than improvement) in subscale reliability if CASP items from other subdomains are ignored. Next, we examine the cross-validation of the CASP’s bifactor representation in an independent sample specific to a patient population, as well as compare CASP’s unidimensional indices across samples.

Findings from the TILDA-W3 sample were mostly similar to those obtained from the initial SHARE-W6. First, the model-comparisons were extended for retention of the CASP BiFactor model. Furthermore, QoL-construct level indices (e.g., *ω*, *ω*_Hier_, H_Rep_, & FD) aligned with results obtained from the previous SHARE-W6 sample. However, specific item-level indices (e.g., ARPB, IECV) were found to be slightly more pronounced in the second TILDA-W3 patient sample. Also, the lower ECV value in the TILDA-W3 sample is further reflected in the difference between CASP_TOT_’s *ω* and *ω*_Hier_ for indexing the reliable variance due to its subdomains. Specifically, in the TILDA-W3 patient sample, *ω*(.93) - (.77)*ω*_Hier_ = .16, indicating that 16% of the reliable variance in CASP_TOT_ scores is due to the subdomains. Further inspection of CASP subscales’ *ω*_Hier_ values affirmed previous findings for inadequate reliable variance after factoring out CASP_TOT_’s general QoL factor. The model-level, construct-unidimensional, and item-level indices are summarized across samples in Table 3 below [15].

**Table 3:** Summary Unidimensionality Indices for CASP by Study Sample

	SHARE W6 Sample	TILDA W3 Sample
*Uni-Dim Indices*		
ECV **/** ECV_New_	.74 / .74	.64 / .64
*ω* **/** *ω*_Hier_	.92 / .80	.93 / .77
H	.92	.92
FD	.96	.92
PUC	.55	.53
ARPB	.11	.44
IECV (# _items_ > .80)	.70 (5_[4,9,10,11,12]_)	.63 (5_[3,4,7,11,12]_)

*Note*. *ECV* estimated common variance

## Discussion

This study examined the widely used CASP-12 QoL measure using IRT to examine the general factor’s robustness to multidimensionality, as well as the usefulness for subdomains’ as narrower individual-differences indicators.

There are several important limitations to the current study that warrant note. First, the extension of our tentative findings from SHARE to TILDA data samples should be viewed cautiously, as we noted substantive compositional differences, such as general / patient populations, respectively. [16] Second, the current psychometric findings for CASP is limited to cross-sectional designs. Future research may extend these findings by assessing a longitudinal extension of the bifactor model presented here, in terms of usefulness for detecting CASP responsiveness; This is a pertinent criterion for evaluating PRO measures [17].

In this first-IRT inspection of CASP’s psychometric properties, the CASP-12’s general QoL factor was found to be well-specified by a bifactor model for specifying subdomains/content homogeneity as sources of nuisance variance. Furthermore, the CASP-12’s total score (general factor) exhibited acceptably high reliability in older populations across both broader community-dwellers, as well as among narrower-patient respondents. In contrast, the CASP-12’s specific subfactors were found to exhibit unacceptably low reliability, suggesting only CASP-12’s global score is currently appropriate for substantive interpretation and meaningful use [18]. Finally, the CASP’s original 12-item measure was identified as-having a potentially useful, 5-item subset for succinct indexing of QoL-unitary scores for future researchers’ use in structural-estimation models.

## Additional file

Additional file 1: **Table S1.** Item -Discrimination (α) and Difficulty (b) Parameter Estimates from CASP-12 BiFactor Model (DOCX 24 kb)

## Footnotes

1 **Secondary** use of **de-i**dentified data information permitted waiver of necessary study ethical review.

2 Please find full-item discriminations and thresholds (locations) parameter estimates for the retained, CASP-bifactor model listed in Additional file 1.
