# Supplementary material for: Bifactor model of the CASP-12’s general factor for measuring quality of life in older patients
Source: J Patient Rep Outcomes. 2018 Dec 4;2:57. doi: 10.1186/s41687-018-0078-x (PMC6279927; doi:10.1186/s41687-018-0078-x)
Supplement: Supplementary file 1 — Table S1. Item -Discrimination (α) and Difficulty (b) Parameter Estimates from CASP-12 BiFactor Model (DOCX 24 kb) [file 41687_2018_78_MOESM1_ESM.docx]

**Online Supplementary Material – APPENDIX A**

The mean relative-bias from forcing CASP-12 data to a unidimensional measurement model was *M* = 12.34%. According to Muthén, Kaplan, and

Hollis (1987), parameter bias of less-than (>) 15% is considered acceptable for limiting substantive distortions in structural equation models [18].

| **Table A-1** | | | | | | | | | | | | | | | | | | | | | | | | | |
| --- | --- | --- | --- | --- | --- | --- | --- | --- | --- | --- | --- | --- | --- | --- | --- | --- | --- | --- | --- | --- | --- | --- | --- | --- | --- |
| ***Item -Discrimination* (*α*) *and Difficulty* (*b*) *Parameter Estimates from CASP-12 BiFactor Model*** | | | | | | | | | | | | | | | | | | | | | | | | | |
|  | | | | | | | | | | | | | | | | | | | | | | | | | |
|  |  | CASP-12 General and Specific Dimensions’ IRT-Parameter Estimates | | | | | | | | | | | | | | | | | | | | | | | |
|  |  | Discriminations | | | | | | | |  |  | Locations | | | | | | | | | | | | | |
| Item |  | ***a*_TotQoL_** | |  | ***a_Con/Aut_*** | |  | ***a_Pleas/SR_*** | |  |  | ***b_1_*** | |  | | | ***b_2_*** | |  | | | ***b_3_*** | | |  |
| 1 |  | -1.44 | (0.02) |  | 0.64 | (.01) |  |  |  |  |  | 2.27 | 0.02 | |  | -0.11 | | 0.02 | |  | -1.66 | | 0.03 |  | |
| 2 |  | -1.38 | (.00) |  | 1.47 | (.00) |  |  |  |  |  | 3.58 | 0 | |  | 0.91 | | 0.00 | |  | -1.27 | | 0.02 |  | |
| 3 |  | -1.58 | (0.02) |  | 1.75 | (.05) |  |  |  |  |  | 4.69 | 0.06 | |  | 2.06 | | 0.03 | |  | -0.04 | | 0.01 |  | |
| 4(r) |  | 1.06 | (0.01) |  | - | - |  | 0.48 | 0.01 |  |  | 0.12 | 0.01 | |  | -1.76 | | 0.01 | |  | -3.45 | | 0.02 |  | |
| 5 |  | -0.23 | (0.03) |  | 0.83 | (.07) |  | - | - |  |  | 2.82 | 0.04 | |  | 0.98 | | 0.02 | |  | -0.3 | | 0.01 |  | |
| 6 |  | -0.7 | (0.12) |  | 0.51 | (.20) |  | - | - |  |  | 1.38 | 0.01 | |  | -0.06 | | 0.01 | |  | -1.14 | | 0.02 |  | |
| 7 |  | 1.21 | (0.01) |  |  |  |  | 1.85 | 0.02 |  |  | -1.06 | 0.01 | |  | -3.59 | | 0.02 | |  | -5.65 | | 0.04 |  | |
| 8 |  | 1.74 | (0.02) |  |  |  |  | 2.49 | 0.03 |  |  | -1.45 | 0.02 | |  | -4.64 | | 0.04 | |  | -7.16 | | 0.06 |  | |
| 9(r) |  | 0.8 | (0.01) |  |  |  |  | 1.26 | 0.02 |  |  | -0.08 | 0.01 | |  | -2.61 | | 0.02 | |  | -4.61 | | 0.03 |  | |
| 10(r) |  | 2.04 | (0.02) |  |  |  |  | 0.87 | 0.01 |  |  | 0.88 | 0.01 | |  | -2.13 | | 0.02 | |  | -4.81 | | 0.03 |  | |
| 11(r) |  | 2.07 | (0.01) |  |  |  |  | 1.35 | 0.01 |  |  | 1.04 | 0.01 | |  | -1.99 | | 0.02 | |  | -4.89 | | 0.03 |  | |
| 12(r) |  | 2.16 | (0.02) |  |  |  |  | 1.45 | 0.01 |  |  | 1.28 | 0.01 | |  | -1.99 | | 0.02 | |  | -4.76 | | 0.03 |  | |
| ***Substantive-Dimensionality Indices***  Relative-Bias *M* (*SD*) Δ Reliability | | | | | | | | | | | | | | | | | | | | | | | | | |
| 12.34% (2.72) Δ .04 | | | | | | | | | | | | | | | | | | | | | | | | | |
| *Note. N* = 63,669. Standard errors reported in parentheses. Linear transformation of α and *b* parameters correspond approximately to item slopes and intercepts in CTT-factor models. ECV = estimated common variance; Relative-Bias is computed as the relative-difference between loadings from the l-Dim and BiFact model (general dimension). Relative-bias is reported as the mean-estimate across items with standard deviations reported in parentheses; 1-Dim reliability = .86 / BiFactor reliability = .90. | | | | | | | | | | | | | | | | | | | | | | | | | |
